# Evaluation of the Chemical and Mechanical Properties of Hardening High-Calcium Fly Ash Blended Concrete

**DOI:** 10.3390/ma8095282

**Published:** 2015-09-07

**Authors:** Wei-Jie Fan, Xiao-Yong Wang, Ki-Bong Park

**Affiliations:** Department of Architectural Engineering, Kangwon National University, Chuncheon-Si 200-701, Korea; E-Mails: fanwjkw@gmail.com (W.-J.F.); kbpark@kangwon.ac.kr (K.-B.P.)

**Keywords:** high calcium fly ash, hydration, kinetic model, calcium hydroxide, strength, concrete

## Abstract

High-calcium fly ash (FH) is the combustion residue from electric power plants burning lignite or sub-bituminous coal. As a mineral admixture, FH can be used to produce high-strength concrete and high-performance concrete. The development of chemical and mechanical properties is a crucial factor for appropriately using FH in the concrete industry. To achieve sustainable development in the concrete industry, this paper presents a theoretical model to systematically evaluate the property developments of FH blended concrete. The proposed model analyzes the cement hydration, the reaction of free CaO in FH, and the reaction of phases in FH other than free CaO. The mutual interactions among cement hydration, the reaction of free CaO in FH, and the reaction of other phases in FH are also considered through the calcium hydroxide contents and the capillary water contents. Using the hydration degree of cement, the reaction degree of free CaO in FH, and the reaction degree of other phases in FH, the proposed model evaluates the calcium hydroxide contents, the reaction degree of FH, chemically bound water, porosity, and the compressive strength of hardening concrete with different water to binder ratios and FH replacement ratios. The evaluated results are compared to experimental results, and good consistencies are found.

## 1. Introduction

Fly ash is produced in coal-burning electricity generation stations and has been widely used for producing high-performance concrete. Fly ashes can be divided into two categories according to their calcium content. The first type of fly ash, containing less than 10% analytical CaO, is generally a product of the combustion of anthracite and bituminous coals. The second type of fly ash, typically containing 15% to 40% analytical CaO, is generally a product of the combustion of lignite and sub-bituminous coals [1].

Many investigations on the physical, chemical, and durability properties of concrete incorporating low-calcium fly ash (FL, CaO less than 10%) have been performed [2,3,4,5,6]. For concrete containing high-calcium fly ash (FH, CaO more than 10%), the investigations are relatively insufficient. Due to the problems associated with the FH chemical compositions (high free CaO and sulfur contents), the suitability of FH as a mineral admixture in the concrete industry is viewed with skepticism. To overcome these problems and to apply FH blended concrete during dam construction, Tsimas and Moutsatsou-Tsima [7] put forward systematic industry methods. Untreated fly ash was cheaply upgraded by grinding at a specially designed ball mill with simultaneous hydration for the reduction of free CaO. When the free calcium oxide content in FH is less than 3.5% and the sulfur content in FH is less than 7%, the soundness, performance, and durability of FH blended concrete can meet code specifications. Antiohos and Tsimas [8] investigated the role of the reactivity of reactive silica in the hydration of FH-cement blends. They found that after the first month of the hardening process, the soluble silica of FH holds a predominant role and that the silica is increasingly dissolved in the matrix, forming additional cementitious compounds with binding properties, principally a second generation calcium silicate hydrate (CSH). Erdogdu and Turker [9] investigated the effects of FH particle size distribution on the strength of FH-cement mortars. They found the reactivity of FH will increase with the increasing fineness and that the strength of FH blended mortar relates closely to the size fractions of FH. Summarily, experimental investigations [7,8,9] show that the properties of FH blended concrete relate to cement hydration and FH reaction. To evaluate the properties of FH blended concrete, both cement hydration and FH reaction should be considered. 

Compared to the abundant experimental investigations on FL blended concrete [7,8,9], theoretical models for FH-cement blended concrete are very limited. Papadakis [10,11,12] proposed a simplified scheme describing the chemical reactions of the FH in FH-cement blends and developed mathematical expressions for predicting the final chemical and volumetric composition of FH blended concrete. Schindler and Folliard [13] described the heat evolution process of FH blended cement using a three-parameter model. The hydration time parameter, hydration shape parameter, and ultimate degree of hydration parameter are adopted for evaluating the hydration of concrete. Saeki and Monteiro [14] formulated a model to predict the reaction between calcium hydroxide and mineral admixtures (including high-calcium fly ash, low-calcium fly ash, and slag). The parameters of the prediction model are dependent on the physical and chemical characteristics of the mineral admixtures. Conversely, there are some limits for current models of FH blended concrete. Papadakis’ model is only valid for hardened concrete and cannot be used to evaluate the properties development of hardening concrete [11,12]. Schindler and Folliard’s model [13] is limited to evaluating the hydration heat of FH blended concrete. The interactions between cement hydration and FH reactions are not considered. Saeki and Monteiro’s model [14] is useful for evaluating the calcium hydroxide content in hydrating cement-FH blends. However, other aspects related to the hydration process, such as chemically bound water, porosity, and compressive strength, cannot be determined using Saeki and Monteiro’s model. 

To overcome the limitations of the current models [11,12,13,14] and to systematically evaluate the chemical and mechanical properties development of FH blended concrete, this paper proposes a numerical model for hydrating cement-FH blends. The proposed model analyzes the cement hydration, the free CaO reaction in FH, and the reaction of phases in FH other than free CaO. The properties of hardening FH blended concrete, such as the reaction degrees of cement and FH, the calcium hydroxide content, chemically bound water, porosity, and compressive strength, can be determined. To improve readability, the summary of abbreviations is shown in Table 1. 

**Table 1:** Summary of abbreviations.

Abbreviations	Text
FH	high-calcium fly ash
FL	low-calcium fly ash
SF	silica fume
H	H_2_O
C	CaO
S	SiO_2_
A	Al_2_O_3_
F	Fe_2_O_3_
$\bar{\text{S}}$	SO_3_
CF	free CaO
CH	Ca(OH)_2_
C_3_S	3CaO·SiO_2_
C_2_S	2CaO·SiO_2_
C_3_A	3CaO·Al_2_O_3_
C_4_AF	4CaO·Al_2_O_3_·Fe_2_O_3_
CSH	calcium silicate hydrate
A–S	aluminosilicate
C–A–S	calcium aluminosilicate
C–A–H	calcium aluminate hydrate
C_4_A_3_$\bar{\text{S}}$	monocalcium aluminosulfate
FHA10%	FH replaces aggregate by 10% weight of cement
FHA20%	FH replaces aggregate by 20% weight of cement
FHA30%	FH replaces aggregate by 30% weight of cement
FHC10%	FH replaces cement by 10% weight of cement
FHC20%	FH replaces cement by 20% weight of cement
FHC30%	FH replaces cement by 30% weight of cement
TG/DTA	thermogravimetry/differential thermal analysis

## 2. Hydration Model of Cement-FH Blends

### 2.1. Hydration Model of Cement

In this study, the cement hydration model originally developed by Tomosawa [15] and modified by Park [16] is adopted to simulate the development of cement hydration. The kinetic processes of cement hydration, such as the formation and destruction of an initial impermeable layer, the activated chemical reaction process, and the following diffusion-controlled process, are considered in the modeling process [15,16]. This model is expressed as a single equation consisting of three coefficients: *k*_d_, the reaction coefficient in the induction period; *D*_e_, the effective diffusion coefficient of water through the CSH gel; and *k*_r_, a coefficient of the reaction rate of cement, as shown in Equation (1):

(1a) $$\frac{d\text{α}}{dt}=\frac{3\left(S_{\text{w}}/S_{\text{0}}\right){\text{ρ}}_{\text{w}}C_{\text{w-free}}}{(v+w_{\text{g}})r_{\text{0}}\rho_{\text{c}}}\frac{1}{(\frac{1}{k_{\text{d}}}-\frac{r_{\text{0}}}{D_{\text{e}}})+\frac{r_{\text{0}}}{D_{e}}{\left(1-\text{α}\right)}^{\frac{-1}{3}}+\frac{1}{k_{\text{r}}}{\left(1-\text{α}\right)}^{\frac{-2}{3}}}$$

(1b) $$k_{\text{d}}=\frac{B_{\text{ce}}}{{\text{α}}^{1.5}}+C_{\text{ce}}{\text{α}}^{3}$$

(1c) $$D_{\text{e}}=D_{\text{e0}}\ln(\frac{1}{\text{α}})$$

where α is the degree of cement hydration; *v* is the stoichiometric ratio by mass of water to cement (=0.25); *w*_g_ is the physically bound water in the CSH gel (=0.15); ρ_w_ is the density of water; *C*_w-free_ is the amount of water at the exterior of the CSH gel; *r*_0_ is the radius of unhydrated cement particles (*r*_0_ = 3/(*S*ρ_c_), in which the terms *S* and ρ_c_ stand for the Blaine surface area and the density of the cement, respectively); *S*_w_ is the effective surface area of the cement particles in contact with water and *S*_0_ is the total surface area if the surface area develops unconstrained; *B*_ce_ controls the rate of the initial shell formation and *C*_ce_ controls the rate of the initial shell decay; and *D*_e0_ is the initial value of the effective diffusion coefficient.

The amount of water in the capillary pores *C*_w-free_ is expressed as a function of the degree of hydration in the previous step, as shown in Equation (1d):

(1d) $$C_{\text{w-free}}=\frac{W_{\text{0}}-0.4*\text{α}*C_{\text{0}}}{W_{\text{0}}}$$

where *C*_0_ and *W*_0_ are the mass fractions of cement and water in the mix proportion.

The effect of temperature on the reaction coefficients is assumed to follow Arrhenius’s law [15,16]. Using the proposed Portland cement hydration model, Tomosawa [15] evaluated the heat evolution rate, chemically bound water, and compressive strength of hardening concrete. Park *et al.* [16] predicted the temperature distribution in ultra-high-strength concrete using this hydration model. A good correlation was found between the analysis results and the experimental results.

### 2.2. Reaction of Free CaO in FH

Cement notation chemistry is used throughout this paper, where H: H_2_O, C: CaO, S: SiO_2_, A: Al_2_O_3_, F: Fe_2_O_3_, $\bar{\text{S}}$: SO_3_, CH: Ca(OH)_2_. For FH, the majority of calcium is present in crystalline and reactive constituents, such as C_3_A, C_4_A_3_$\bar{\text{S}}$, C$\bar{\text{S}}$, free CaO, and calcium aluminosilicate (C–A–S) glass. Free CaO has both negative and positive effects on concrete performance. Free CaO can harm the volume stability and the concrete durability [1]. Conversely, some researchers state that a moderate amount of free CaO (3.5% free CaO) is essential for the initial activation of high calcium fly ash [7]. The free CaO is hydrated rapidly to CH as follows [11]:

C + H → CH
(2)

Chen [17] studied the hydration kinetic process of free CaO in systems containing high-calcium fly ash-H_2_O at different curing temperatures. The reaction of free CaO in FH is a first-order reaction whose rate is directly proportional to the degree of reaction. Chen [17] proposed that the reaction degree of free CaO α_CF_ can be described as follows:

(3) $${\text{α}}_{\text{CF}}(t)=1-\frac{1}{e^{kt}}$$

where *k* is reaction rate coefficient (*k* is 0.09/h when the temperature is 20 °C, and the dependence of the reaction rate coefficient on the curing temperature can be described using Arrhenius’s law [17]). 

### 2.3. Reaction of Phases in FH other than Free CaO

FH essentially consists of aluminosilicate glass modified by the presence of large amounts of calcium and magnesium. High-calcium fly ashes are pozzolans that will react with the calcium hydroxide resulting from the hydration of Portland cement to produce calcium silicate hydrate (CSH) and calcium aluminate hydrate (C–A–H). However, FH are also hydraulic in nature, as they will react directly with water to form a range of hydration products, and a mix of FH and water will set, harden, and gain a certain strength even in the absence of Portland cement (or other activators). This behavior results from the hydraulic nature of the crystalline products and the dissolution and reaction of the glass due to the presence of soluble alkalis and calcium within the FH [10,11,12]. Therefore, high-calcium fly ash has both hydraulic properties and pozzolanic properties.

Saeki and Monteiro [14] proposed that the reaction between FH and calcium hydroxide is a diffusion-controlled process. The reaction rate of FH relates to the available calcium hydroxide content in a cement-FH system. Giergiczny [18] investigated the hydraulic activity of FH using an isothermal heat evolution experiment. Similar to cement, the reaction process of an FH-water paste consists of an initial dormant period, a phase boundary reaction process, and a diffusion process. Papadakis [12] proposed that similar to the control of Portland cement, the strength of FH blended concrete relates to the calcium silicate hydrate (CSH) content. Similar to cement, the reaction products of high-calcium fly ash adhere to the surfaces of the remaining FH particles. Therefore, in this paper, similar to that of Portland cement, the reaction of FH is assumed to consist of three processes: an initial dormant period, a phase boundary reaction process, and a diffusion process. The reaction equations of FH are originally proposed as follows:

(4a) $$\frac{d{\text{α}}_{\text{FH}}}{dt}=\frac{m_{\text{CH}}(t)}{P}\frac{W_{\text{cap}}}{W_{\text{0}}}\frac{3{\text{ρ}}_{\text{w}}}{v_{\text{FH}}r_{\text{0FH}}{\text{ρ}}_{\text{FH}}}\frac{1}{(\frac{1}{k_{\text{dFH}}}-\frac{r_{\text{0FH}}}{D_{\text{eFH}}})+\frac{r_{\text{0FH}}}{D_{\text{eFH}}}{\left(1-{\text{α}}_{\text{FH}}\right)}^{\frac{-1}{3}}+\frac{1}{k_{\text{rFH}}}{\left(1-{\text{α}}_{\text{FH}}\right)}^{\frac{-2}{3}}}$$

(4b) $$k_{\text{dFH}}=\frac{B_{\text{FH}}}{{{\text{α}}_{\text{FH}}}^{1.5}}+C_{\text{FH}}{{\text{α}}_{\text{FH}}}^{3}$$

(4c) $$D_{\text{eFH}}=D_{\text{e0FH}}\ln(\frac{1}{{\text{α}}_{\text{FH}}})$$

where α_FH_ is the reaction degree of the active part in FH other than free CaO, *m*_CH_(*t*) is the mass of calcium hydroxide, *W*_cap_ is the mass of capillary water, *P* is the quality of the FH in the mixing proportion other than the mass of free CaO, *ν*_FH_ is the stoichiometry ratio by mass of CH to FH other than free CaO, *r*_0FH_ is the radius of the FH particle, ρ_FH_ is the density of FH, *k*_dFH_ is the reaction rate coefficient in the dormant period (*B*_FH_ and *C*_FH_ are coefficients), *k*_rFH_ is the reaction rate coefficient of the phase boundary reaction process, and *D*_e0__FH_ is the initial diffusion coefficient. 

Papadakis [11] proposed that FH consists of an active part and an inert part. The active part includes an aluminosilicate glass phase, a calcium phase, and a sulfate phase. The reacted ratio of the active part of FH other than free CaO can be calculated according to Equation (4a) through (4c). The crystalline part of alumina found in corundum and A_3_S_2_ will not react. The non-reactive part of silica is present in quartz and in crystalline A–S phases. The inert part is assumed to be chemically inert and does not react. The reacted ratio of FH other than free CaO (including both an active part and an inert part) can be obtained based on Equation (4a) through (4c) and the mass compositions of the fly ash. Considering both the active part and the inert part, the total reaction degree of FH other than free CaO can be determined as follows:

(5) $${\text{α}}_{\text{FH-total}}={\text{γ}}_{\text{active}}\times {\text{α}}_{\text{FH}}$$

where α_FH-total_ is the reactive degree of the total FH other than free CaO and γ_active_ is the weight fraction of the active part of FH other than free CaO.

### 2.4. Mutual Interactions among Cement Hydration, the Reaction of Free CaO in FH, and the Reaction of Phases in FH other than Free CaO

As proposed by Papadakis [11], the chemical reactions of the mineral compounds of Portland cement can be expressed as follows:

2C_3_S + 6H → C_3_S_2_H_3_ + 3CH (6a)

2C_2_S +4H → C_3_S_2_H_3_ + CH (6b)

(6c) $${\text{C}}_{3}\text{A}+\text{C}\bar{\text{S}}{\text{H}}_{2}+10\text{H}\to {\text{C}}_{4}\text{A}\bar{\text{S}}{\text{H}}_{12}$$

C_4_AF + 2CH + 10H → C_6_AFH_12_ (6d)

FH has a composition closer to Portland cement than low-calcium fly ash (FL) or silica fume (SF). The active part of FH consists of an aluminosilicate glass phase, a calcium phase, and a sulfate phase. The majority of the active part of the silica in FH is present in aluminosilicate (A–S) and calcium aluminosilicate (C–A–S) glass. Calcium silicate hydrate (CSH) is formed from the pozzolanic action of the reactive silica of FH as follows [11]:

2S + 3CH → C_3_S_2_H_3_ (7a)

Part of alumina, found as tricalcium aluminate (C_3_A) and monocalcium aluminosulfate (C_4_A_3_$\bar{\text{S}}$), reacts with water, CH, and/or gypsum, as in cement hydration, showing these high early strengths. This reaction in an excess of sulfate ions (as applies in this case) can be totally described as follows [11]:

(7b) $${\text{C}}_{3}\text{A}+\text{C}\bar{\text{S}}{\text{H}}_{2}+10\text{H}\to {\text{C}}_{4}\text{A}\bar{\text{S}}{\text{H}}_{12}$$

The crystalline part of alumina found in corundum and A_3_S_2_ will not react. The rest of the alumina present in A–S and C–A–S glass is expected to react as follows [11]:

(7c) $$\text{A}+\text{C}\bar{\text{S}}{\text{H}}_{2}+3\text{CH}+7\text{H}\to {\text{C}}_{4}\text{A}{\text{H}}_{12}$$

The Portland cement and FH can be analyzed in terms of oxides: total CaO (C), free CaO (CF), SiO_2_ (S), Al_2_O_3_ (A), Fe_2_O_3_ (F), SO_3_ ($\bar{\text{S}}$), and other oxides or impurities denoted by R. Let *fi,*c and *fi,*p denote the weight fractions of the constituent *i* (*i* = C, CF, S, A, F, $\bar{\text{S}}$, R) in the cement and FH other than free CaO, respectively. Let γ_S_ and γ_A_ denote the active fraction of the oxides S and A in the FH, respectively. Considering the production of calcium hydroxide from cement hydration and the reaction of free CaO in FH, the consumption of calcium hydroxide from the reaction of phases in FH other than free CaO, and the content of calcium hydroxide in FH-cement blends can be determined as follows:

(8a) $$CH\text{(}t\text{)}=C_{\text{0}}RCH_{\text{CE}}\text{α}+1.321CF\text{(}t\text{)}-v_{\text{FH}}{\text{α}}_{\text{FH}}P$$

(8b) $$v_{\text{FH}}=(1.851{\text{γ}}_{\text{S}}f_{S,p}+2.182{\text{γ}}_{\text{A}}f_{\text{A,p}}\text{)}-1.321(f_{\text{C,p}}-0.7f_{\bar{\text{S}}\text{,p}}\text{)}$$

(8c) $$CF(t)=CF_{\text{0}}{\text{α}}_{\text{CF}}(t)$$

where *RCH_CE_* is the produced calcium hydroxide from 1 gram of cement, *CF*(*t*) is the mass of the reacted free CaO at time *t*, and *CF*_0_ is the mass of the free CaO content in FH. The term *C*_0_*RCH*_CE_α considers the production of calcium hydroxide from cement hydration, the term 1.321*CF*(*t*) considers the production of calcium hydroxide from the reaction of free CaO in FH, and the term *−ν*_FH_α_HF_*P* considers the consumption of calcium hydroxide from the reaction of phases in FH other than free CaO.

Similarly, the mass of calcium silicate hydrate (CSH) relates to the cement hydration and FH reaction. The mass of CSH can be determined as follows:

(8d) $$\text{C}SH\text{(}t\text{)}=2.85f_{\text{S,c}}\text{α}C_{\text{0}}+{\text{γ}}_{\text{S}}f_{\text{S,p}}\alpha_{\text{FH}}P\text{)}$$

where 2.85*f*_S,__c_α*C*_0_ considers the production of CSH from cement hydration, and 2.85γ_S_*f*_S,p_α_FH_*P* considers the production of CSH from the FH reaction.

The mass of chemically bound water relates to the cement hydration, the reaction of free CaO in FH, and the reaction of phases in FH other than free CaO. The mass of chemically bound water *W*_cbm_ can be determined as follows:

(8e) $$W_{\text{cbm}}=v\times C_{\text{0}}\times \text{α}+RCW_{\text{FH}}\times {\text{α}}_{\text{FH}}\times P+0.321CF(t)$$

where *RCW*_FH_ is the mass of chemically bound water from 1 gram of reacted FH (*RCW*_FH_ = 0.09 [11]). The term *ν × C*_0_ *×* α denotes the production of chemically bound water from cement hydration, the term 0.321*CF*(*t*) denotes the production of chemically bound water from the reaction of free CaO in FH, and the term *RCW*_FH_ *×* α_FH_ *× P* denotes the production of chemically bound water from the reaction of phases in FH other than free CaO. 

The mass of capillary water relates to the cement hydration, the reaction of free CaO in FH, and the reaction of phases in FH other than free CaO. The mass of capillary water *W*_cap_ can be calculated as follows:

(8f) $$W_{\text{cap}}=W_{\text{0}}-0.4\times C_{\text{0}}\times \text{α}-RCW_{\text{FH}}\times {\text{α}}_{\text{FH}}\times P-0.321CF(t)-RPW_{\text{FH}}\times {\text{α}}_{\text{FH}}\times P$$

where *RPW*_FH_ is the mass of physically bound water from 1 gram of reacted FH (*RPW*_FH_ = 0.15 [11]). The term *RPW*_FH_ *×* α_FH_ *× P* denotes the consumption of physically bound water from the reaction of phases in FH other than free CaO.

The porosity of hydrating blends is reduced due to the Portland cement hydration, the reaction of free CaO in FH, and the reaction of phases in FH other than free CaO. The porosity, ɛ, can be estimated as follows:

(8g) ε=WρW−Δεc−ΔεCF−ΔεFH

where Δɛ_c_, Δɛ_CF_, and Δɛ_FH_ are the porosity reduction due to the Portland cement hydration, the reaction of free CaO in FH, and the reaction of phases in FH other than free CaO, respectively; these terms can be determined from the amount of chemically bound water consumed in the Portland cement hydration, the reaction of free CaO in FH, and the reaction of phases in FH other than free CaO, respectively [11].

### 2.5. Summary of the Proposed Hydration Model of Cement-FH Blends

Papadakis [11] proposed a chemical-based steady-state model that can be used to evaluate the final chemical and volumetric composition of concrete containing high-calcium fly ash. However, on a construction site, the workers and designers are interested in not only the final properties of the FH concrete but also the evolution of the properties of the FH concrete over time. In this paper, by combining Papadakis’ chemical-based steady-state model and the kinetic reaction mechanisms involved in cement hydration and FH reaction, a kinetic model is uniquely proposed to describe the hydration process of FH concrete. (The mathematical equations shown in Section 2.3 and Section 2.4 are our original work.) The FH reactions are treated separately from the Portland cement, and some interactions are taken into account through the free water content and the calcium hydroxide content. The proposed model considers the influence of the water to binder ratio, the FH to cement ratio, the mineral compositions and the particle size of cement and FH, and the curing conditions on hydration. Based on the degree of hydration, the development of early-age properties and the durability aspect of FH concrete can be predicted. 

**Figure 1:**
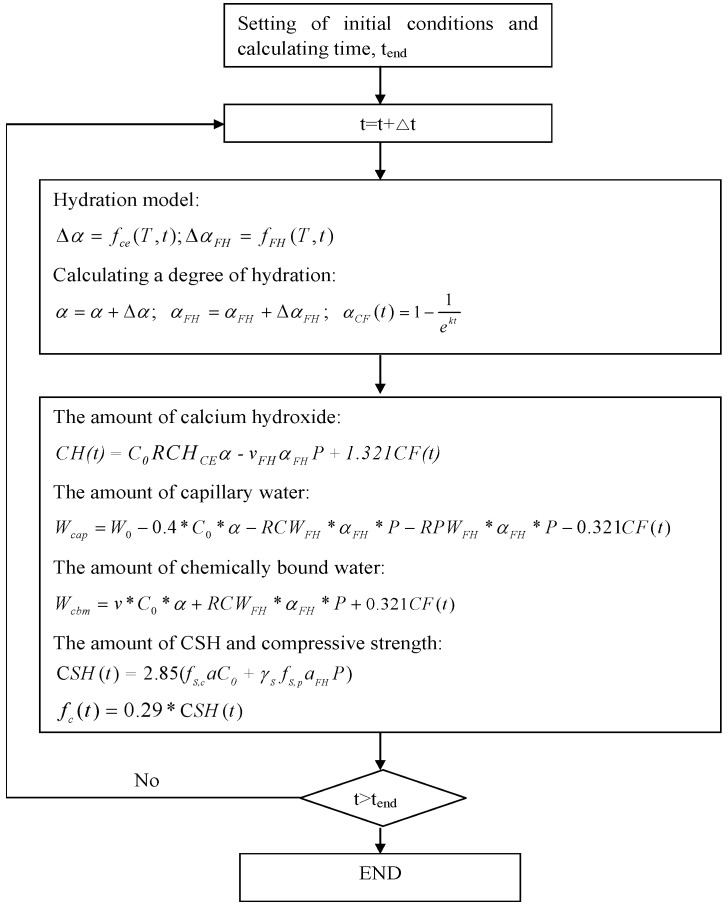
The flow chart for a numerical simulation process.

The flow chart for a numerical simulation process is shown in Figure 1. The proposed model considers the influences of the Portland cement hydration, the reaction of free CaO in FH, and the reaction of phases in FH other than free CaO on the property developments of cement-FH blends. Using the degree of hydration of cement α, the reaction degree of free CaO in FH α_C__F_, and the reaction degree of phases in FH other than free CaO α_FH_ at each time step, the amount of calcium hydroxide, capillary water, chemically bound water, porosity, CSH, and compressive strength can be calculated. 

## 3. Verification of the Proposed Model

Experimental results from reference [11] are used to verify the proposed model. Papadakis [11] studied property developments of high-calcium fly ash blended mortars. A rapid-setting Portland cement was used (400 m^2^/kg Blaine’s fineness). Prior to use, high-calcium fly ash was pulverized to meet the cement mean particle size. The physical properties and chemical compositions of cement and FH are shown in Table 2. The total CaO content in FH is 23%, the free CaO content in FH is 5%, and the glass content in FH is 50%. The mixture proportions are given in Table 3. In the control specimen, the water-cement ratio (*W*/*C*) was 0.5 and the aggregate-cement ratio (*A*/*C*) was 3. Two different cases were studied: FH replaces either aggregate or cement. When FH replaces aggregate, three contents of FH were selected: 10%, 20%, and 30% additions to the control cement weight for specimens FHA, 10%, FHA, 20%, and FHA, 30%, respectively. When FH replaces the cement, the same FH contents were selected: 10%, 20%, and 30% replacements of the control cement weight for specimens FHC, 10%, FHC, 20%, and FHC, 30%, respectively.

The developments of strength, porosity, bound water, and calcium hydroxide were measured [11]. The specimens for the strength measurements were prisms of 40 × 40 × 160 mm, cured under lime-saturated water at 20 °C and tested after 3, 14, 28, 49, 91, 182, and 364 days. FH-cement pastes were also prepared, representing the paste matrix of the mortar specimens, and analyzed for CH content, chemically bound water content, and porosity at 3, 7, 14, 28, 49, 112, 182, and 364 days after casting and moist curing [11]. For each test, the moist-cured paste specimen was stripped, placed in a pre-weighed glass mortar, and pulverized. The mortar with the material was placed in an oven at 105 °C until the weight was constant. These weight indications were used to determine the chemically bound water content and porosity. To determine the CH content, a paste sample of the oven-dried powder was examined by a combination of quantitative X-ray diffraction analysis and TG/DTA (thermogravimetry/differential thermal analysis). The mass loss corresponding to the decomposition of Ca(OH)_2_ occurs between 440 °C and 520 °C. The paste properties were reduced to the corresponding mortar specimen volume by multiplying the appropriate paste volume/mortar volume fraction [11].

**Table 2:** Physical and chemical characteristic of cement and FH.

Properties	Cement	FH
Physical properties	-	-
BET specific surface (m^2^/g)	1.3	6.2
Particle mean diameter (μm)	14.0	12.6
Density (kg/m^3^)	3130	2660
Chemical analysis (%)	-	-
SiO_2_	20.10	39.21
Al_2_O_3_	4.25	16.22
Fe_2_O_3_	3.49	6.58
CaO	63.20	22.78 (5.18 free)
SO_3_	2.88	4.3
LOI	0.86	2.10

**Table 3:** Mixing proportions of the specimens.

Specimens	Cement (kg/m^3^)	Water (kg/m^3^)	FH (kg/m^3^)	Aggregate (kg/m^3^)	Water to Binder Ratio	FH to Cement Ratio	Aggregate to Cement Ratio
control	514.6	257.4	0	1543.8	0.5	0	3
FHA, 10%	514.6	257.4	51.5	1492.1	0.46	0.1	2.9
FHA, 20%	514.6	257.4	102.9	1440.5	0.42	0.2	2.8
FHA, 30%	514.6	257.4	154.4	1388.8	0.39	0.3	2.7
FHC, 10%	463.1	257.4	51.5	1536.0	0.5	0.11	3.32
FHC, 20%	411.7	257.4	102.9	1528.3	0.5	0.25	3.71
FHC, 30%	360.2	257.4	154.4	1520.5	0.5	0.43	4.22

### 3.1. Evaluation of Calcium Hydroxide Contents 

In the proposed model, the reaction of free CaO and other phases in FH are simulated separately. Equation (3) is used to model the rapid reaction of free CaO in FH, and Equation (4) is used to model the reaction of phases in FH other than free CaO. Using the chemical analysis of FH shown in Table 2, we can determine the weight fractions of S, A, C, and $\bar{\text{S}}$ in the FH other than free CaO. Furthermore, the active part of FH other than free CaO, γ_active_, which includes an aluminosilicate glass phase, a calcium phase, and a sulfate phase, can be calculated. The weight fractions of FH used for modeling are shown in Table 4.

Using the experimental results of calcium hydroxide from Portland cement and a predictor-corrector algorithm [19], the reaction coefficients of Portland cement *B*_ce_, *C*_ce_, *D*_e0_, and *k*_r_ can be determined. Using the experimental results of calcium hydroxide from cement-FH paste, the FH reaction coefficients *B*_FH_, *C*_FH_, *D*_e0__FH_, and *k*_rFH_ can be determined. The values of the reaction coefficients are shown in Table 5. The fit parameters for a material are not changed from one mix to the other. For concrete with different water to binder ratios or FH replacement ratios, the reaction coefficients of cement and FH do not change.

**Table 4:** Weight fractions of FH.

γ_S_	*f*_S, p_	γ_A_	*f*_A, p_	$f_{\bar{\text{S}},\text{p}}$	*f*_C, p_	CaO_free_
0.7	0.413	0.7	0.1711	0.0453	0.1856	0.0518
γ_active_ = γ_S_*f*_S, p_(1−CaO_free_) + γ_A_*f*_A, p_(1−CaO_free_) + *f*_C, p_(1−CaO_free_) + $f_{\bar{\text{S}},\text{p}}$ (1−CaO_free_)						0.61

**Table 5:** Coefficients of the reaction model.

*B*_ce_ (cm/h)	*C*_ce_ (cm/h)	*k*_r_ (cm/h)	*D*_e0_ (cm^2^/h)
1 × 10^−8^	0.81	2.33 × 10^−6^	3.79 × 10^−10^
*B*_FH_ (cm/h)	*C*_FH_ (cm/h)	*k*_rFH_ (cm/h)	*D*_e0__FH_ (cm^2^/h)
2.22 × 10^−11^	0.08	5.45 × 10^−7^	2 × 10^−8^

After calibrating the coefficients of the reaction model, multiple checks, such as the evolution of chemically bound water, porosity, CSH contents, and compressive strength, were performed to verify the proposed model. The procedure of the proposed model is similar to that of Tomosawa *et al.* [15,19]. In Tomosawa’s model, the heat evolution of Portland cement was adopted to calibrate the coefficients of the hydration model. Furthermore, the evolution of the mechanical properties in early-age concrete was described using functions of the degree of hydration [15,19].

**Figure 2:**
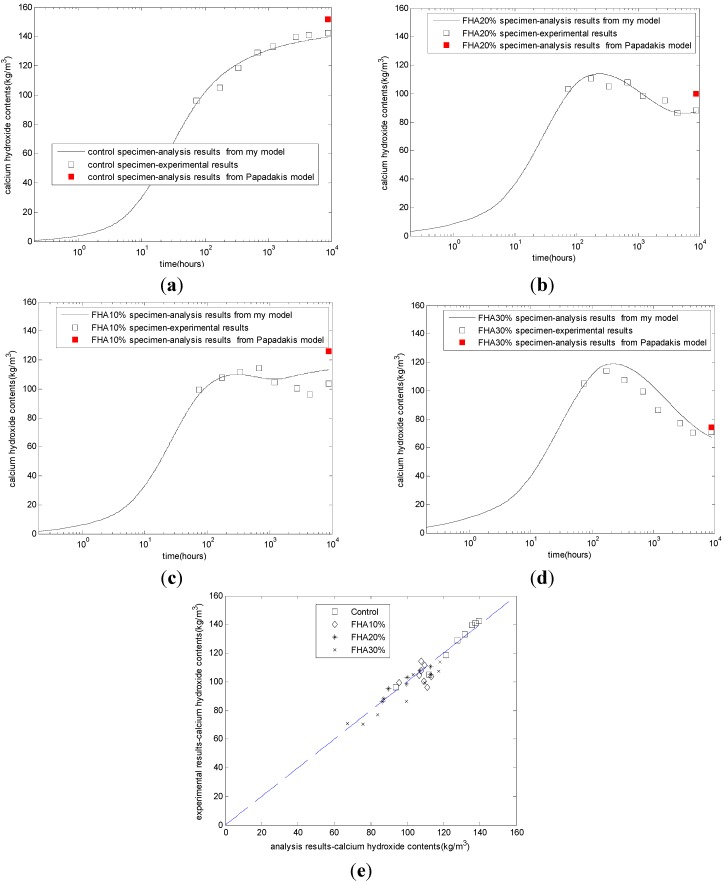
Development of calcium hydroxide contents. (**a**) Control concrete; (**b**) FHA, 20% concrete: FH replaces aggregate by 20% weight of cement; (**c**) FHA, 10% concrete: FH replaces aggregate by 10% weight of cement; (**d**) FHA, 30% concrete: FH replaces aggregate by 30% weight of cement; (**e**) comparison between analysis results and experimental results.

The evaluation results of the calcium hydroxide contents are shown in Figure 2. The CH content of the control specimen increases with time until a steady state is attained (Figure 2a). The CH content of FHA specimens presents a rather complicated picture due to the simultaneous CH production from the reaction of cement and the free CaO and CH consumption from other phases of the FH. In the first week, due to the rapid free CaO hydration, the CH contents are higher in the fly ash specimens (Figure 2b–d) than that of the control specimen (Figure 2a), These CH contents pass through a maximum due to higher CH production but decrease afterward as the FH-CH reaction proceeds at higher rates. At a late age, with the FH replacing ratio increasing from 10% to 30%, the CH contents decrease correspondingly. For concrete with 10% FH (Figure 2c) and 20% FH (Figure 2b), at the age of one year, the calcium hydroxide will slightly increase. This result may be due to the depletion of the active part of FH. Because the proposed model has modeled the rapid production of calcium hydroxide from the free CaO reaction in FH, the production of calcium hydroxide from cement hydration, and the consumption of calcium hydroxide from other phase reactions in FH, the proposed model can describe the complicated evolution process of cement-FH blends. Figure 2e shows the comparison between the experimental results and the analysis results for different mixing proportions. The correlation coefficient between the experimental results and the analysis results is 0.95. 

In addition, Figure 2 shows the analysis results of calcium hydroxide from Papadakis’ model [11]. Papadakis assumed that at the age of 365 days, the reaction rate of cement and FH is very slow. The age of 365 days can be approximately regarded as the steady state age of FH blended concrete. As shown in Figure 2, Papadakis’ model can calculate the final CH content. However, because Papadakis’ model does not consider the kinetic reaction process of FH, this model cannot calculate the evolution of CH in cement-FH blends. The calculation result of Papadakis’ model is a point, not a curve. Conversely, the calculation result from our kinetic hydration model is a curve.

**Figure 3:**
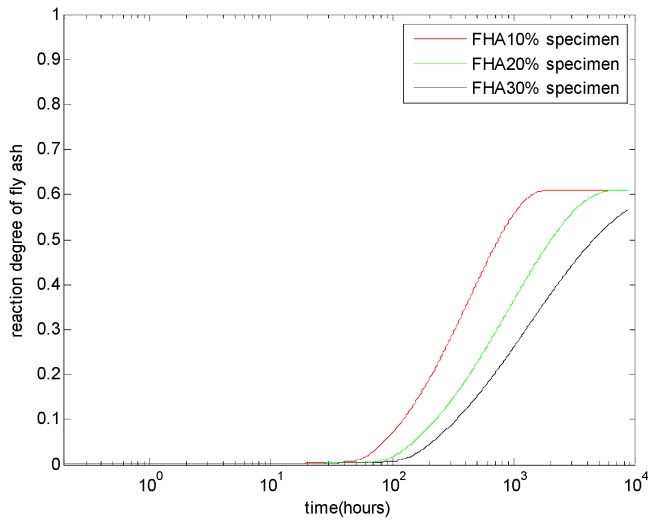
Reaction degree of high-calcium fly ash with different FH contents: FH replaces aggregate by 10%, 20%, and 30% weight of cement and a water to binder ratio of 0.5.

The evaluation of the reaction degree of fly ash α_FH-total_ is shown in Figure 3. As shown in this figure, given a certain water to binder ratio, with an increasing replacement level of fly ash, the alkaline-activating effect of the cement would be weaker, so that the reactivity of the fly ash decreases [20]. Conversely, at a late age, for concrete with 10% and 20% FH, the active part of FH has totally reacted (the ultimate reaction degree equals the weight fraction of the active part γ_active_). For concrete with 30% FH, until the age of one year, part of FH is still un-reacted.

### 3.2. Evaluation of Chemically Bound Water and Porosity

**Figure 4:**
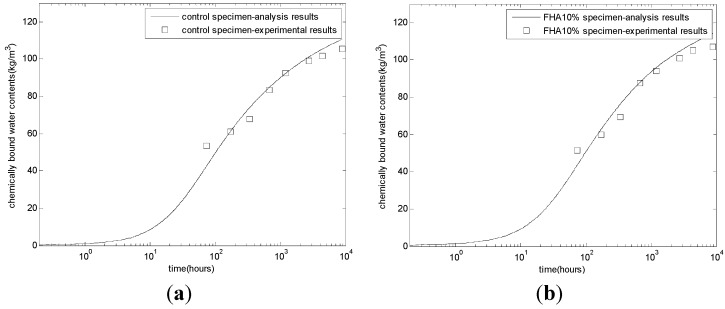

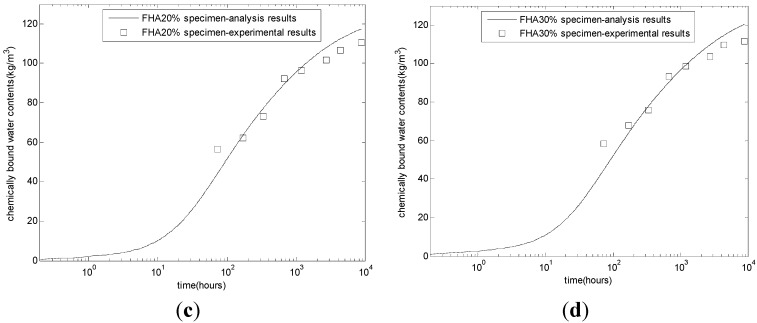
Development of chemically bound water contents. (**a**) Control concrete; (**b**) FHA10% concrete: FH replaces aggregate by 10% weight of cement; (**c**) FHA20% concrete: FH replaces aggregate by 20% weight of cement; (**d**) FHA30% concrete: FH replaces aggregate by 30% weight of cement.

**Figure 5:**
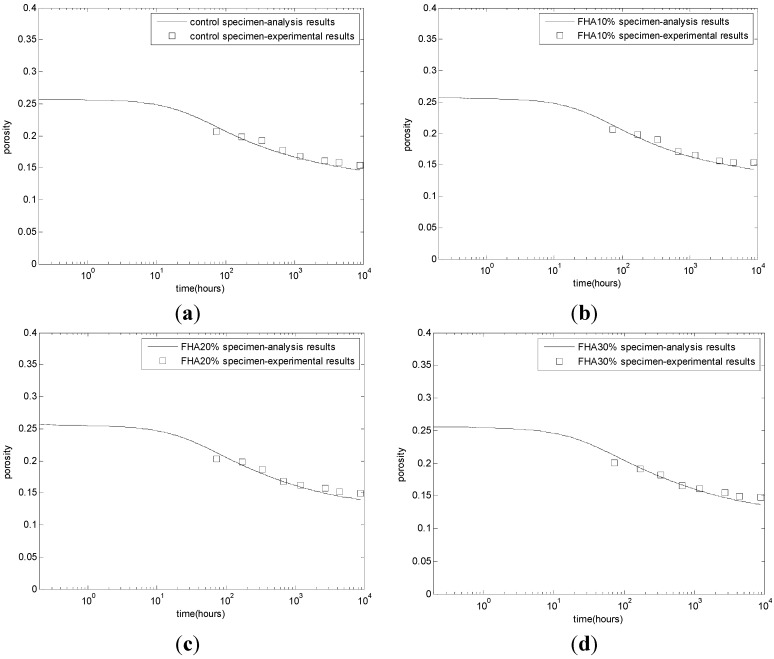
Development of porosity contents. (**a**) Control concrete; (**b**) FHA10% concrete: FH replaces aggregate by 10% weight of cement; (**c**) FHA20% concrete: FH replaces aggregate by 20% weight of cement; (**d**) FHA30% concrete: FH replaces aggregate by 30% weight of cement.

The evaluation results of chemically bound water and porosity contents are shown in Figure 4 and Figure 5, respectively. A higher chemically bound water (H) content (Figure 4) and a lower porosity (Figure 5) is observed for all of the FHA specimens compared to the corresponding control values from the initiation of the cement hydration. This increase in H values or the similar decrease in porosity values is almost proportional to the fly ash content in the specimen. This behavior can be partly explained as being due to the presence of free CaO in the FH and the hydration of the tricalcium aluminate present in FH. The early formation of water-rich and pore-filling ettringite and its subsequent transformation to other hydrates contributes significantly to the increased hydration and decreased porosity [11].

### 3.3. Evaluation of Compressive Strength

**Figure 6:**
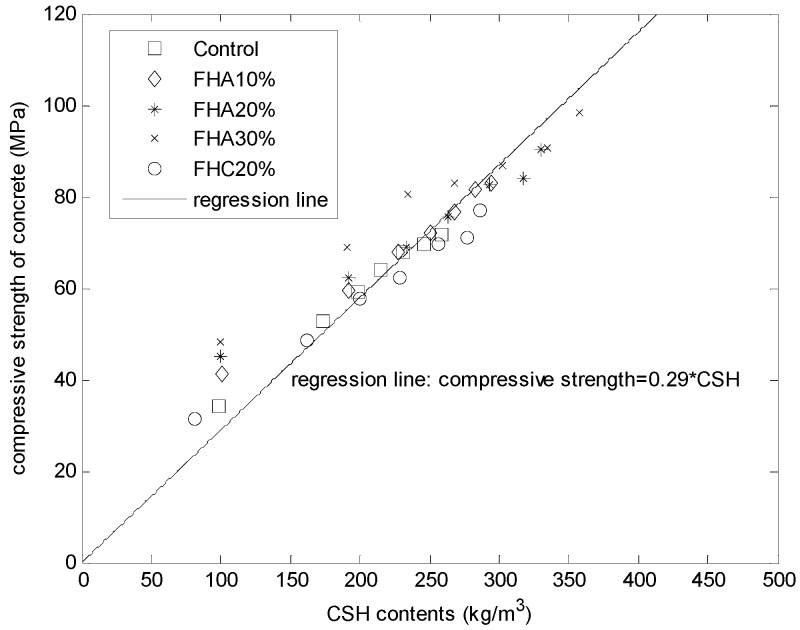
Compressive strength of mortars as a function of calculated (CSH) content.

Papadakis [11,12] proposed that the compressive strength of cement-based materials, such as concrete containing low-calcium fly ash, high-calcium fly ash, and slag, can be estimated from the CSH content. Using the proposed hydration model considering both cement hydration and FH reaction (Equation (8d)), we can calculate the CSH contents for mortars with different FH contents, such as the control specimen, FHA, 10% (FH replaces aggregate by 10% weight of cement), FHA, 20% (FH replaces aggregate by 20% weight of cement), FHA30% (FH replaces aggregate by 30% weight of cement), and FHC, 20% (FH replaces cement by 20%). Figure 6 presents the compressive strength of the FH blended mortars as a function of the calculated CSH contents. As shown in Figure 6, for concrete with different FH contents (control, FHA, 10%, FHA, 20%, FHA, 30%, and FHC, 20%) and at different ages (3, 14, 28, 49, 91, 182, and 364 days), a single linear relationship exists between the compressive strengths and the calculated CSH amounts, *i.e.*, the compressive strength = 0.29 × CSH. 

**Figure 7:**
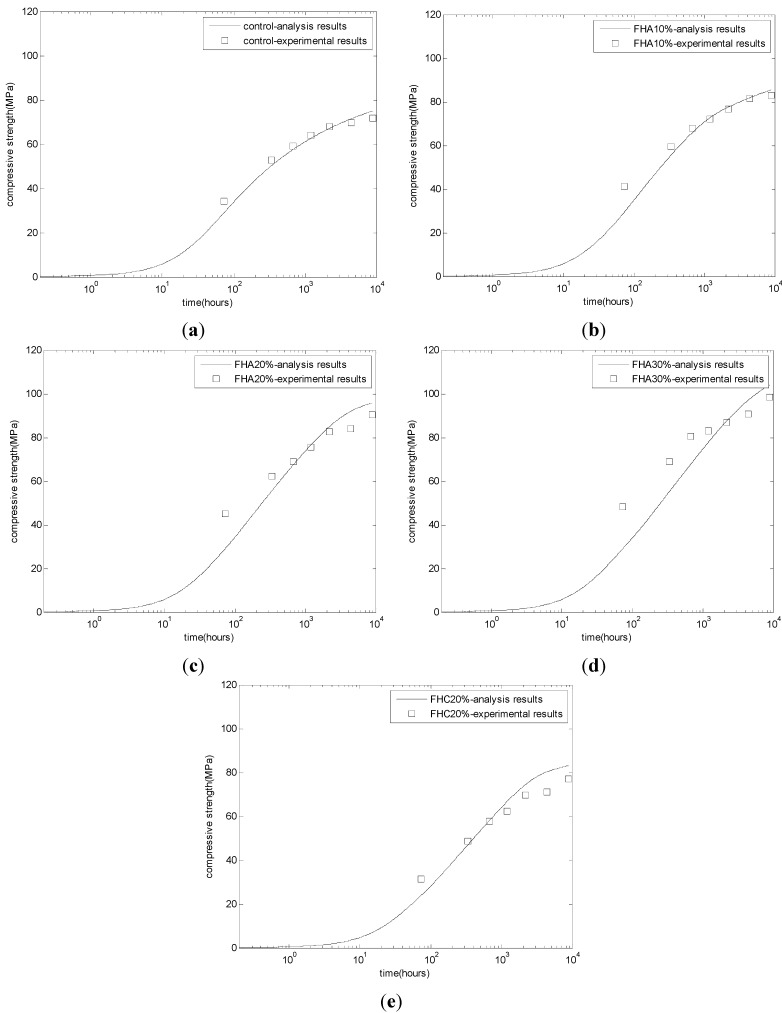
Evaluation of the compressive strength of FH concrete. (**a**) Control concrete; (**b**) FHA, 10% concrete: FH replaces aggregate by 10% weight of cement; (**c**) FHA, 20% concrete: FH replaces aggregate by 20% weight of cement; (**d**) FHA, 30% concrete: FH replaces aggregate by 30% weight of cement; (**e**) FHC, 20% concrete: FH replaces cement by 20%.

Using the regressed linear relationship between the compressive strength and the CSH content, we can evaluate the development of the compressive strength of FH-cement blends. Figure 7 shows the comparison between the experiment results and the simulation results of the compressive strength. For mortar containing FH-replacing aggregate (FHA mortars, as shown in Figure 7b–d), the compressive strength of FHA specimens is higher than that of the control specimen (Figure 7a) since the early ages. However, for mortar containing FH-replacing cement (FHC mortar, as shown in Figure 7e), at early ages, the compressive strength of the FHC specimen is lower than that of the control specimen (Figure 7a), and at late ages, due to the evolution of the FH reaction, the compressive strength of the FHC specimen surpasses that of the control specimen. Conversely, in the early age of three days, the simulation value is slightly lower than the experimental value. This result may be due to the omission of some factors, such as the contribution of ettringite from the FH reaction [11] and the nucleation effects of FH [11]. For FHA, 30% concrete (Figure 7d), due to the abundant ettringite produced from the FH reaction, at the early ages of three and 14 days, the analysis results show more deviations from the experimental results than for the other mixing proportions. 

In addition, the free CaO in FH also affects the compressive strength developments of hardening cement-FH blends. Tsimas and Moutsatsou-Tsima [7] investigated the influence of free CaO contents on the compressive strength development. When the free CaO content increases from 1.8% to 3.5%, the compressive strength will increase correspondingly. This result is mainly due to the stimulus of the free CaO on the initial FH reaction [7]. However, when the free CaO content further increases from 3.5% to 6.8%, the soundness of the concrete will be impaired and the compressive strength will decrease. Therefore, the free CaO presents double-edged effects on the strength development. For the model proposed in this paper, further investigations should be performed regarding FH with various chemical compositions and free CaO contents.

**Figure 8:**
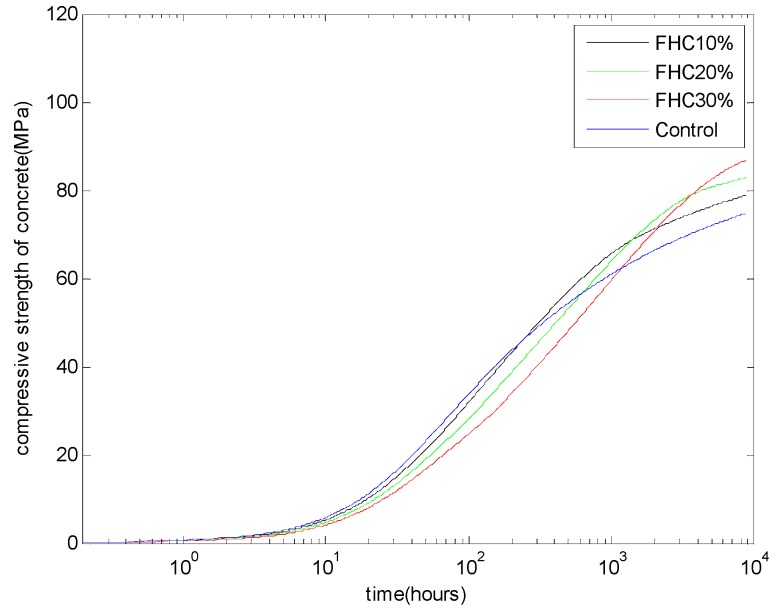
Compressive strength development of FH concrete: water to binder ratio of 0.5 and FH replaces cement by 10%, 20%, and 30%.

In the concrete industry, considering the economic and environmental effects, high-calcium fly ash is generally used as a mineral admixture to replace cement. Using the hydration model, we can calculate the CSH contents. Furthermore, using the regressed linear relationship between the compressive strengths and CSH amounts, we can calculate the compressive strength development of FH concrete. Figure 8 shows the analysis results of the compressive strength of FH blended concrete with a water to binder ratio of 0.5 and different FH contents: FH replacing cement by 10%, 20%, and 30%. The proposed model can reproduce the compressive strength crossover phenomenon between the control Portland cement concrete and the FH blended concrete. With increasing FH replacement ratios, the reactivity of FH will decrease (as shown in Figure 3) and the age corresponding to the crossover of the compressive strength will be postponed. 

**Figure 9:**
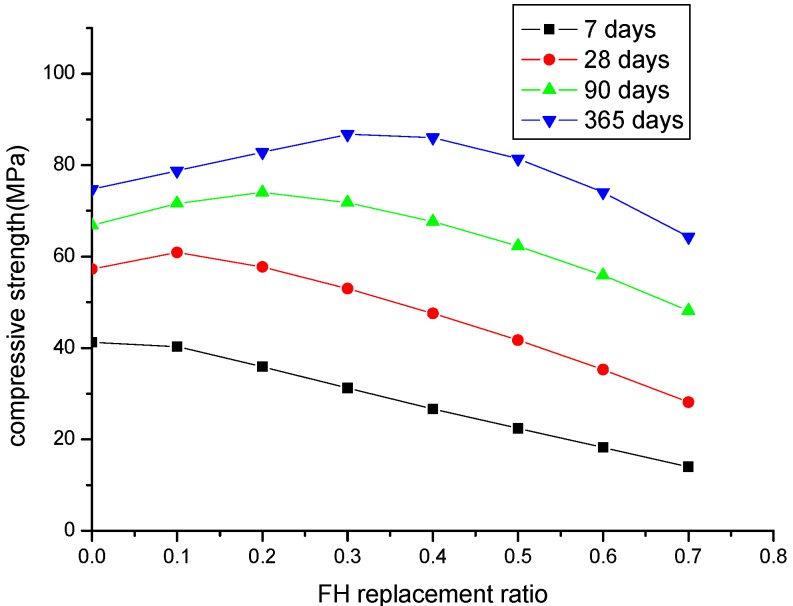
Parameter analysis of the compressive strength of concrete: a water to binder ratio of 0.5 and FH replaces cement by 10% to 70%.

Figure 9 shows the compressive strength development of FH blended concrete with different FH contents (FH replaces cement by 10% to 70%) at different ages of seven days, 28 days, 90 days, and 365 days. At an early age of seven days, compared to the control concrete, the compressive strength of FH concrete almost linearly decreases with increasing FH content. With increasing curing age, due to the development of the FH reaction, the compressive strength of FH concrete will surpass that of the control concrete. However, for concrete incorporating larger FH contents (more than 50% cement is replaced by FH), due to the reduction of FH reactivity, at the age of one year, the compressive strength of FH concrete is still lower than that of the control concrete. Therefore, 50% can be regarded as the maximum replacement ratio of FH. Over this maximum replacement ratio, the compressive strength of FH blended concrete will be lower than that of the control concrete. Conversely, at the age of one year, when the FH replacement ratio is higher than 30%, the compressive strength of concrete will decrease. Therefore, 30% FH can be regarded as an optimum FH content for concrete incorporating FH as a cement-replacing mineral admixture. The optimum FH content can be explained as follows: according to Equation (8d), the CSH content produced from the FH reaction, 2.85γ_S_*f*_S,p_α_FH_*P*, relates to the FH content *P* and the reaction degree of FH α_FH_. With increasing FH content *P*, the reactivity of FH will decrease (as shown in Figure 3), and therefore, there is a peak value for the product of FH content *P* and the reaction degree of FH α_FH_. The FH content corresponding to the peak value can be regarded as the optimum replacement of FH.

## 4. Conclusions

(1) This paper proposes a numerical procedure to simulate the hydration of cement and high-calcium fly ash (FH) blends. The hydration of cement, the reaction of free CaO in FH, and the reaction of phases in FH other than free CaO are modeled. The interactions among the hydration of cement, the reaction of free CaO in FH, and the reaction of phases in FH other than free CaO are considered through the available calcium contents and capillary water contents in the system. The reaction of free CaO in FH is described as a first-order reaction. The reaction of other phases in FH is divided into three processes, *i.e.*, an initial dormant period, a phase boundary reaction process, and a diffusion process. The reaction coefficients of other phases in FH are obtained from experimental results of calcium hydroxide in cement-FH blends. The fit parameters for a material are not changed from one mix to the other. For concrete with different water to binder ratios or FH replacement ratios, the reaction coefficients of cement and FH do not change.

(2) Using the numerical procedure, the hydration degree of cement, the reaction degree of free CaO in FH, and the reaction degree of phases in FH other than free CaO are calculated. The amount of calcium hydroxide, bound water, porosity, and compressive strength of FH blended concrete are determined considering the contributions from the hydration of cement, the reaction of free CaO in FH, and the reaction of phases in FH other than free CaO. At different curing ages, for FH blended concrete with different FH contents, a single linear relationship exists between the compressive strengths and the calculated CSH amounts. The proposed model can reproduce the compressive strength crossover phenomenon between control Portland cement concrete and FH blended concrete. The proposed model also can be used to determine the optimum content of FH. 
